# Mr. Torrence, on Scarlatina Anginosa

**Published:** 1810-05

**Authors:** W. H. Torrence


### To Dr. BRADLEY.

Dear Sir,

HAVE the goodness to insert in your next Medical and Physical Journal, the following account of Scarlatina Anginosa, as it has appeared in this country during the greater part of last winter. My principal object in this short memoir is, to draw the attention of practitioners to the great advantages to be derived from cold affusion in the treatment of that disease. Some of your Readers may be pleased to hear an account of this epidemic as it appeared in the neighbourhood of Johnny Grott's House.

---

### *On SCARLATINA ANGINOSA.*

By Mr. W. H. Torrence.

NO description of this disease, which has fallen into my hands, applies so perfectly to that form of it which appeared in this county in the end of the year 1809, and beginning of 1810, as that given in the Lectures of the late learned and indefatigable Professor Burserius de Kanifield. Sydenham, indeed, has given, in his usual masterly manner, a history of the phenomena of one form of this disease, the Scarlatina Simplex; but it does not appear that this accurate historian of disease, and minute observer of symptoms, ever had an opportunity of observing ing it under the form of scarlatina anginosa, otherwise, it is not to be believed that so prominent a symptom as the affection of the throat would have escaped his notice.

He describes the disease as one of the mildest in its nature, requiring for its management little more than the common attentions to rest and quiet, and seems to fear worse consequences from the "nimia medici diligentia," than from the effects of the disease itself.

Doctor Cullen, it is well known, considered scarlatina and cynanche maligna as different diseases, and accordingly, in his nosological arrangement, has placed the latter in the order Phlegmasiæ, and not in that of Exanthemata. Such distinction, however, seems now to be very generally abandoned.

"It seems highly probable that these are both names of the same distemper, with some little variety in a few symptoms; and this opinion is confirmed by our finding that they are both epidemical at the same time." Vid. Heberden's Commentaries, cap. 7, § 2.

During the prevalence of the present epidemic, I have had repeated and almost daily opportunities of ascertaining the identity of these two forms of disease, and am satisfied that they stand in the same relation to each other, as the distinct does to the confluent small-pox, differing only in degree, and both deriving their origin from the same contagion.

Sydenham observes, that this disease commonly makes its appearance in autumn. In this instance, however, the epidemic first shewed itself about the close of winter, or at least did not become frequent till about the middle of January. One solitary case, indeed, I first visited on the 28th of October, 1809. It is to be observed, however, that the winter in this country was more than usually mild, and, in many respects, bore a considerable resemblance to the weather and temperature which commonly prevail in the end of autumn.

In most cases, the disease was ushered in with the usual symptoms of pyrexia, mild however in degree. Slight rigors, or at least some sense of cold, most frequently in the back and loins; head-ache; sometimes a degree of vertigo, were complained of. The tongue at first little changed from its natural and healthy appearance; but as the disease advanced in progress, it assumed a whitish cream-like covering. The pulse was not greatly accelerated; urine scanty, and of a deep brown colour.

On the third, fourth, or fifth day, the scarlet efflorescence cence made its appearance, most commonly first on the face, neck, or chest, to which parts, in some cases, it was altogether confined. In others, however, it gradually extended itself over the whole body and limbs, giving the whole person, in some instances, a pretty close resemblance in colour to that of a boiled lobster. The colour, on pressure with the hand, immediately disappeared, leaving the skin remarkably white, but, on removing the pressure, it almost as immediately returned.

In the course of two or three days, the colour began to fade, and a mealy or branny desquamation of the cuticle took place. At this stage of the disease, a slight degree of œdema of the face or ancles sometimes occurred.

Such was the history of the milder cases of this epidemic; and in such cases, I have no doubt the patient was safe, even in spite of the "nimia medici diligentia." In other cases, however, a more formidable train of symptoms was to be combated. The common symptoms of pyrexia, at the commencement of the disease, were attended by great dejection of mind, and apprehensions of death; deep seated pain at the back part of the head; stiffness and rigidity of the muscles of the neck; a sense of fullness in the throat; difficult and painful deglutition; pains in all the limbs; great lassitude and inaptitude to motion, indicated the approach of the worst form of the disease. In such cases the pulse was feeble and greatly increased in frequency, while the countenance exhibited, by the shrunk and flaccid features, that fear of dissolution which so frequently occupied the mind of the patient. The skin in every case was hot, but not hard, and in some the heat, as measured by the thermometer, was so high as 106° of Fahrenheit.

On examining the throat, the tonsils were found considerably swelled; in some covered with small grey coloured specks, but in others with brown coloured sloughs, discharging a sanies of most offensive odour. In several instances which fell under my observation, this slouging extended itself from the amygdalæ into the meatus auditorii, through the Eustachian tube, discharging the same offensive sanies from the external aperture of the ears. In one unfortunate boy, whom I attended, I believe the organs of hearing to be completely destroyed by the cause. In other cases the sloughing proceeded from the velum pendulum palati into the passage to the nose, and a similar offensive sanies was discharged from the nostrils.

The

The practice I followed in the treatment of this disease, was extremely simple, and I have the satisfaction to say very successful.

By referring to my notes of this epidemic, I find I have attended seventy-three cases, all of which have terminated favourably. Of this number sixty-five were subjected to the affusion of cold sea-water; which was done by placing the patient on a low seat, in the middle of a large tub, and pouring the water, to the quantity of at least two English gallons, on the head and shoulders, so as to flow all over the body and limbs of the patient. The patient was then wiped dry, and immediately put to bed. The affusion was repeated as often as the return of severe head-ache and increasing heat of surface seemed to render adviseable, seldom oftener than morning and evening during the continuance of fever.

The uniform effect of the affusion was a decrease of fever, relief from distressing head-ache and pain of the limbs, followed soon after by quiet and refreshing sleep. So fully satisfied were my patients of the efficacy of this seemingly harsh measure, and so sensible of the relief they experienced from it, that it was not unusual for them to have recourse to this powerful remedy on their own accord, on the re-accession of heat and febrile uneasiness.

Of the sixty-five cases treated with the cold affusion, the disease in nineteen was completely cut short on the second or third day of its duration; in all of them, the advantages derived from it were obvious and decided. Depression of spirits was succeeded by the cheering influence of hope; head-ache and febrile heat of surface were removed, and the patient looked forward to a recovery, of which no doubt was entertained.

The remaining eight of my patients were either in so advanced a period of the disease before I first visited them, or laboured under such affections of the chest, as rendered the propriety of this treatment doubtful. In some of these, however, tepid bathing was used with very decided advantage.

In the application of the cold affusion, the very judicious and pointed directions of the late Dr. Currie, to whom medical science owes so much, were strictly attended to; "*when there was no sense of chilliness present, when the heat of the surface was steadily above what is natural, and when there was no general or profuse perspiration.*"

Thus the repeated use of the cold affusion, the application of a stimulating epithem to the throat, occasionally a blistering blistering plaster, attention to the state of the alvine discharge, as recommended by Dr. Hamilton, in his Observations on Purgative Medicines, constituted almost the whole of my practice in this epidemic. No bark was given in any stage of the disease; but in some of the worst cases, a little wine was recommended during the period of convalescence.

I take leave to remark here, that those gargles, so much recommended by many physicians, were, in bad cases, totally inadmissible. No gargle, whether in the common manner, or thrown in by means of a syringe, could be used without the most imminent danger of suffocation. I found, however, an excellent substitute in the following simple formula.

℞. Acet. distillat. ℥ij. Aq. fervent. ℥iv. M. et inhaletur vapor, medii et grati caloris, more solito.

It was remarkable that anasarcous swellings more frequently supervened after mild cases of this disease, than after such as in their progress had assumed a more formidable appearance. These swellings, however, readily yielded to a few doses of calomel and jalap.

If this short memoir shall have the effect of calling the attention of practitioners to the use of this powerful remedy in scarlatina anginosa, my object will be fully answered.

*Thurso, County of Caithness,*
*April* 6, 1810.

